# DNA methylation cooperates with H3K9me2 at HCN4 promoter to regulate the differentiation of bone marrow mesenchymal stem cells into pacemaker-like cells

**DOI:** 10.1371/journal.pone.0289510

**Published:** 2023-08-29

**Authors:** XiaoLin Sun, Kai Jin, Xiangwei Ding, Zhongbao Ruan, Pei Xu

**Affiliations:** 1 Department of Cardiology, The Affiliated Taizhou People’s Hospital of Nanjing Medical University, Taizhou, Jiangsu, The People’s Republic of China; 2 Department of Haematology, The Affiliated Taizhou People’s Hospital of Nanjing Medical University, Taizhou, Jiangsu, The People’s Republic of China; University of South Florida, UNITED STATES

## Abstract

Sick sinus syndrome (SSS) is a a life-threatening disease, and biological pacemakers derived from bone marrow mesenchymal stem cells (BMSCs) have practical clinical applications. Previous studies demonstrated that epigenetics plays an important role in the differentiation of BMSCs into pacemaker-like cells. However, the underlying mechanisms remain unclear. In the present study, we investigated the role of DNA methylation and histone methylation in pacemaker cells formation and found that changes in DNA and H3K9 methylation occur in the promoter region of the pacemaker cell-specific gene HCN4. In addition, the combined addition of methylation inhibitors was able to improve the efficiency of transduction of Tbx18 in inducing the differentiation of BMSCs into pacemaker-like cells. In vitro experiments have shown that inhibition of DNA methylation and H3K9 methylation can enhance the activity of the HCN4 promoter activity, and both can affect the binding of the transcription factor NKx2.5to the HCN4 promoter region. Further research on the interaction mechanism between DNA methylation and H3K9me2 in the HCN4 promoter region revealed that the two may be coupled, and that the methylesterase G9a and DNMT1 may directly interact to bind as a complex that affects DNA methylation and H3K9me2 regulation of HCN4 transcription. In conclusion, our studies suggest that the mutual coupling of DNA and H3K9 methylation plays a critical role in regulating the differentiation of BMSCs into pacemaker-like cells from the perspective of interactions between epigenetic modifications, and combined methylation is a promising strategy to optimise pacemaker-like cells for in vitro applications.

## Introduction

Slow arrhythmias are a group of arrhythmia manifested mainly by a slowing of the heart rate, and electronic pacemakers are now the standard treatment for slow arrhythmias [1]. Electronic pacemakers are widely used but still have many limitations, such as infection, metal allergy, electrode dislocation, electronic interference and lack of responsiveness to neurohormones [2]. Therefore, biological pacemakers based on somatic cell gene transfection, cell fusion or cell transplantation offer a new option for the treatment of slow arrhythmias. Due to the uncertainty of targeted transfection and transfection efficiency in simple gene biopsies, researchers have gradually turned to cell-based biological pacemaker therapy. For example, mesenchymal stem cell (MSC) transplantation has been shown to improve cardiac function in damaged hearts by regenerating cardiomyocytes (CMs), promoting angiogenesis, reducing cardiac fibrosis and preventing host cardiomyocyte apoptosis [3].

At present, the method of inducing stem cell differentiation into cardiomyocyte-like cells (pacemaker-like cells, atrial-like cells, ventricular-like cells)includes gene transfection, chemical induction and cell co-culture. There are many inducers of stem cell differentiation into cardiomyocytes, such as bone morphogenetic protein, transforming growth factor beta, basic fibroblast growth factor, dimethyl sulfoxide, retinoic acid, 5-azacytidine (5-Aza) [4], but they can also lead to uncontrollable cell differentiation and have not been able to induce single pacemaker-like cells. Gene transfection avoids these problems. As a result, transfection of key genes into pacemaker-like cells has become an effective choice, such as transfection of hyperpolarization-activated cyclic nucleotide-gated Cation Channel 4 (HCN4) to generate pacemaker-like currents [5–9]. However, direct overexpression of the HCN4 gene has been problematic due to poor outcome. In recent years, transfection of some embryonic transcription factors (e.g. the T-BOX family) that regulate cell development from upstream signaling pathways of sinus node development, has resulted in biological pacemakers with physiological functions very similar to those of the sinus node. Previous studies have indicated that expression of the transcription factor TBX18 is capable of initiating differentiation of BMSCs into biological pacemaker-like cells [10]. In summary, there are many methods to induce the differentiation of stem cells into pacemaker-like cells, but they all suffer from low induction efficiency, low cell membrane action potential, and high operational complexity and cost, which are the main reasons limiting the current promotion of biological pacemakers.

Recently, there is accumulating evidence indicating that epigenetic factors play an important regulatory role in cell division and proliferation during cellular and embryonic differentiation. It mainly affects gene transcription without altering DNA sequences, and includes various forms of DNA methylation, histone modifications and regulation of non-coding RNAs [11, 12]. A large number of genomic loci are demethylated during cardiac development [13], and 5-Aza treatment to inhibit DNA methylation or knockdown DNMT3A and 3b has been shown to affect the expression of myocardial fibrosis-related genes [14], while related transcription factors can induce the differentiation of MSCs into cardiomyocyte-like cells through DNA methylation by regulating GATA4 [15]. Histone methylation acts on promoters and enhancers to control transcriptional activity through activation and repression. Methylation of H3K4, H3K36, and H3k79 is associated with gene activation, whereas H3K9, H3K27, and H4K20 are associated with gene silencing [16]. Genetic knockout or BIX01294 treatment to inhibit the histone methyltransferase G9a can induce the expression of cardiac embryonic lineage cell-related genes such as Brachyury and Mesp1in MSCs [17], and our previous study also found that disruption of G9a and KDM3A had an opposite effect on the differentiation of MSCs into cardiomyocytes [18]. The above results indicate a potential role of epigenetic modifications in the differentiation of rat MSCs into cardiac pacemaker-like cells.

In addition, DNA methylation and histone modifications generally interact to regulate transcription, for example, histone modifications can loosen the binding of DNA to histones and generally promote transcription, whereas tightly coiledDNA and histone binding inhibits gene expression [19, 20]. We therefore focus on the role of DNA and histone methylation in the differentiation of MSCs into pacemaker-like cells and the mechanisms of co-regulation between them. Further understanding of these complex epigenetic regulations will improve the efficiency of pacemaker-like cell induction and lay the foundation for clinical applications of biological pacemakers.

## Materials and methods

### Isolation and culture of MSCs

BMSCs were isolated and cultured using the whole bone marrow adherence method. All procedures involving the care and use of animals conformed to the Animal Research: Reporting of In Vivo Experiments (ARRIVE) guidelines and were approved by the laboratory animal management and experimental animal ethics committee of Yangzhou University.

Bone marrow from 8–12 week old, male or female, Wistar rats were collected for isolation of BMSCs. The femurs were removed and placed in a small aseptic beaker and then transferred to a super clean bench. After washing with PBS, the bilateral mummification ends in the femur were resected. A syringe was used to aspirate the cell culture fluid to flush the bone marrow cavity, which was repeated 3–5 times. The cells were resuspended in Dulbecco’s modified Eagle’s medium: nutrient mixture F-12 (DMEM/F12; Gibco) supplemented with 10% fetal bovine serum (FBS; Gibco), 100 IU/ml penicillin, and 100 IU/ml streptomycin, after which the cells were seeded into flasks and cultured in an incubator at 37°C, 5% CO_2_, and 100% relative humidity. After 48 hours, the medium was changed to remove the suspended cells. The medium was then changed every 3 days. When Cells reached 80–90% confluence, 0.25% trypsin containing 0.53 mM EDTA was used to digest the cells. We have previously observed the morphology of cells and detected their surface markers, which were consistent with the characteristics of BMSCs. They have been published in relevant articles [21, 22]. The BMSCs were then expanded and used for *in vitro* and *in vivo* experiments at passages 3–5.

### Treatment of BMSCs with 5-Aza and BIX01294

The BMSCs were divided into five groups: 1) transfected with Tbx18 overexpression vector; 2) transfected with overexpression Tbx18 vector and treatment with 10 μM 5-Aza (Sigma-Aldrich; Merck KGaA); 3) transfected with Tbx18 overexpression vector and treatment with 1 μM BIX01294 (MedChemExpress, Inc.); 4) transfected with Tbx18 overexpression vector and treated with 10 μM 5-Aza and 1 μM BIX01294; 5) no treatment as blank control. All groups of were treated with BIX01294 and/or 5-Aza for one day and then replaced with normal medium. The cells were cultured until day 10, and after digestion and centrifugation, each group of cells was collected for subsequent treatment.

### Plasmid construction and transfection

TBX18 was transduced into BMSCs using recombinant adenovirus as in a previous study [23]. Based on the core active region of the HCN4 promoter (267bp upstream of the TSS) identified by previous studies [24], the three highest scoring potential transcription factors we predicted online via the JASPAR (https://jaspar.genereg.net/), their promoter core region-Basic vectors corresponding to the deletion binding site were constructed, and sent them to Genecreat biological to construct the recombinant vectors. For luciferase assays, HCN4 promoter core fragments were cloned into the pGL3-basic vector.

According to the sequence of the CDS region of the gene provided by NCBI, the CDS of NKx2.5, DNMT1, DNMT3A and DNMT3B were synthesised was synthesized and cloned into pcDNA3.1 vector, G9a CDS sequence was synthesised and cloned into pcDNA3.1-Myc vector. The sh-G9a lentiviral interference vector was obtained from a previous study in our laboratory [18]. The DNMT1-shRNA Plasmid was obtained from Origene (Locus ID 84350).

### Total DNA methylation analysis

Genomic DNA was isolated using TIANamp Genomic DNA Kit (TIANGEN) according to the manufacturer’s instructions. Methylcytosine was quantified in DNA preparations from 5-Aza-treated cultures on days 0–21 using the MethylFlash™ Global DNA 5-mC ELISA Easy Kit (Epigentek). The amountof 5-methylcytosine DNA was quantified by comparison with a standard curve.

### Protein isolation and Western blot

Proteins were extracted from the cells using RIPA lysis buffer containing 1%PMSF (C1055, Pulilai BioTech) to prevent protein degradation. Protein concentrations were measured using the BCA method (CW0014, CWBIO). The protein samples (10 μg/lane) were then separated using 10% SDS-PAGE and electrophoresed. After electrophoresis, the proteins were transferred to PVDF membranes. Membranes were cut according to the markers and incubated in TBS-0.05% Tween 20 containing 5% non-fat milk for 1 h on a shaker at room temperature to block non-specific protein binding. Primary antibodies against H3K9me2(1:1,000; 39239, Active Motif), H3K9me3(1:1,000; 39765, Active Motif), H3K27me3 (1:1,000; 39765, Active Motif), H3 (1:3,000; H0164, Sigma-Aldrich), H3K27me1 (1:1,000; 61016, Active Motif), H3K4me3(1:1,000; 39060, Active Motif), H3K36me3 (1:1,000; 61902, Active Motif), β-actin (1:1,000; SAB3500350; Sigma-Aldrich), cTnT (1:1,000; 21652-1-AP; ProteinTech Group), HCN4 (1:1,000; 55224-1-AP; ProteinTech Group), G9a (1:1,000; sc-515726; Santa Cruz), DNMT3B (1:1,000; sc-81252; Santa Cruz), DNMT3A (1:1,000; sc-52921; Santa Cruz), DNMT1(1:1,000; sc-271729; Santa Cruz), Myc (1:3,000; SAB4301136; Sigma-Aldrich) were incubated with the membranes overnight at 4°C and then washed three times for 10 min in PBST before incubation with the corresponding secondary antibodies (1:3,000, A9169; 1:5,000, AP160P; both Sigma-Aldrich). Positive bands were detected by chemiluminescent reactions (Millipore). Densiometric analysis was performed using Bio-Rad Laboratories software version 3.0.

### Bisulphite treatment and Methylation Specific PCR (MSP)

Potential CpG islands in the promoter region were predicted byMethPrimer(http://www.urogene.org/cgibin/methprimer/methprimer.cgi), and three amplification primers were designed based on the predictions therein. HCN4-1 (forward) 5’-TTTGTTTATAGTTTTTTGGTTATAGTT-3’, (reverse) 5’- TCCCAAAA TTTTCCTAAACCAC-3’; HCN4-2 (forward) 5’-TGTTTATAGTTTTTTGGTTAT AGTT-3’, (reverse) 5’-ACTCCCAAAATTTTCCTAAACC-3’, HCN4-3 (forward) 5’- TTGTTTATAGTTTTTTGGTTATAGTT-3’, (reverse)5’-TCCCAAAATTTTCCTAAA CCAC-3’. The extracted cellular DNA was treated with bisulfate (DP215-02, TIANGEN). The recovered DNA was specifically amplified using the above primers according to the MSP kit (EM101, TIANGEN). The PCR products were gel extracted and the recovered product was ligated to the T vector, then transformed into DH5abacteria and positive colonies were taken for sequencing. At least 10 separate clones were selected for sequencing. The sequencing results were compared with the original sequences using the QUMA (Quantification tool for Methylation Analysis) website(quma.cdb.riken.jp/top/index.html) to generate methylation site maps.

### RNA isolation, quantitative real-time PCR

Total RNA was extracted from the BMSCs of different treatment groups and cardiomyocytes using TRIzol^®^ (DP424, TIANGEN) and cDNA was synthesised from sample RNA using the FastKing RT Kit (KR116, TIANGEN) following the manufacturer’s instructions. The cDNA was then amplified using gene-specific primers and a SYBR Green dye kit (FP313, TIANGEN). The primers used are listed in Table 1. The analysis of relative mRNA expression was performed using the 2^-ΔΔCt^ method, and β-actin was used as an endogenous housekeeping gene to normalize the mRNA levels.

**Table 1:** Primers used for qRT-PCR and plasmid construction.

Gene name	Primer (5’-3’)
HCN4	F: CAGCCAGAAAGCAGTGGA
	R: ATCAGCAACAGCATCGTCA
α-actin	F: CCAACCGTGAGAAGATGAC
	R: GTCGCCAGAATCCAGAAC
cTnT	F: TTCGACCTGCAGGAAAAGTT
	R: GTGCCTGGCAAGACCTAGAG
β-actin	F: CTTCCTGGGTATGGAATCCT
	R: TCTTTACGGATGTCAACGTC
DNMT1	F: GAGTCTGTGGAGAAACCTG
	R: CCTGTCATCTTCCTGTTCAC
DNMT3A	F: GGCAGAATAGCCAAGTTCAG
	R: GAAATGCTGGTCTTTGCCC
DNMT3B	F: CCAGCACTTTAATCTGGCC
	R: ACCATGGCTTTCTCCAGAG
G9a	F: GCTACCATGACTGCGTTCTG
	R: TCCCGGCAGATGATCTNTCTC

HCN4, Hyperpolarization Activated Cyclic Nucleotide-Gated Cation Channel 4.

### Dual luciferase reporter assay

The Dual-Luciferase Reporter Assay System was used to monitor the promoter activity of HCN4. BMSCs were seeded in 24-well plates and transiently cotransfected with 1000 ng of SV40 plasmids and the respective reporter constructs (1:30). Luciferase activity of cell lysate was analyzed using the Dual Luciferase Reporter Assay System (Promega). The firefly luciferase signal was normalized against the Renila signal, and the empty vector PGL3-basic was used as a control.

### Chromatin immunoprecipitation (ChIP)-qPCR

Add formaldehyde (16%) to each sample to cross-linked protein-DNA complex. ChIP experiments were performed using a ChIP assay kit (17–295, MilliporeSigma). After cross-linking, the DNA was fragmented by sonication, which consisted of 5 sec with 10 sec intervals in a cycle lasting 5 min to cool down. Then, protein-DNA complexes were then precipitated with an anti-NKx2.5 antibody (sc-365207, Santa Cruz) and an anti-H3K9me2 antibody (39239, Active Motif). The DNA was extracted using a DNA purification kit (DP214, TIANGEN) and quantified using qPCR. The primers were shown in Table 2. In these experiments, “input” was used to indicate the total protein, the percentage of its protein content bound by the IgG antibody (HA1001, HUABIO) was used as the “control” and the percentage of protein bound by the target antibody was used as the “experimental group”.

**Table 2:** ChIP-qPCR primer sequence.

Name	Primer (5’-3’)
HCN4-P1 (-750-1000bp)	F: CCCTAAATGGCTCCTCTATGGA
	R: TATTAGAAGCCACAGCCGGG
HCN4-P2 (-500-750bp)	F: GGGCAGAGTGTACCCAGATC
	R: TCATTCAGGCCCCAGTTTCC
HCN4-P3 (-250-500bp)	F: CTTGGACCCACGTGATGTCA
	R: GTGTCATCTTCCCCAGGCTC
HCN4-P4 (0-250bp)	F: TGTTGTGTTCCAGGTTGCCT
	R: GGCGACAGACATGGGAGAAT

HCN4, Hyperpolarization Activated Cyclic Nucleotide-Gated Cation Channel 4.

### Co-immunoprecipitation assay

Collected cells were lysed in lysis buffer based on Pierce™ Immunoprecipitation Kit (26149 Thermo Scientific). The cell lysates were then centrifuged, and the supernatant was used for the following experiments. Anti-DNMT1 or anti-Myc magnetic beads were incubated with the cell lysate overnight at 4°C. After magnetization, the immunoprecipitation beads were washed with lysis buffer and resuspended in 1× SDS sample buffer, then heated at 95°C for 5 minutes. The proteins were separated by SDS-PAGE and transferred to NC membrane for Western blotting.

### Statistical analysis

All data were analyzed using the Prism 8.0 (GraphPad). The results are presented as the mean ± standard deviation. Statistical significance was assessed with Student’s t test or analysis of variance (ANOVA) with Tukey’s post hoc test. A p-value <0.05 was considered to be statistically significant.

## Results

### The key epigenetic factors involved in the differentiation of BMSCs

**Fig 1:**
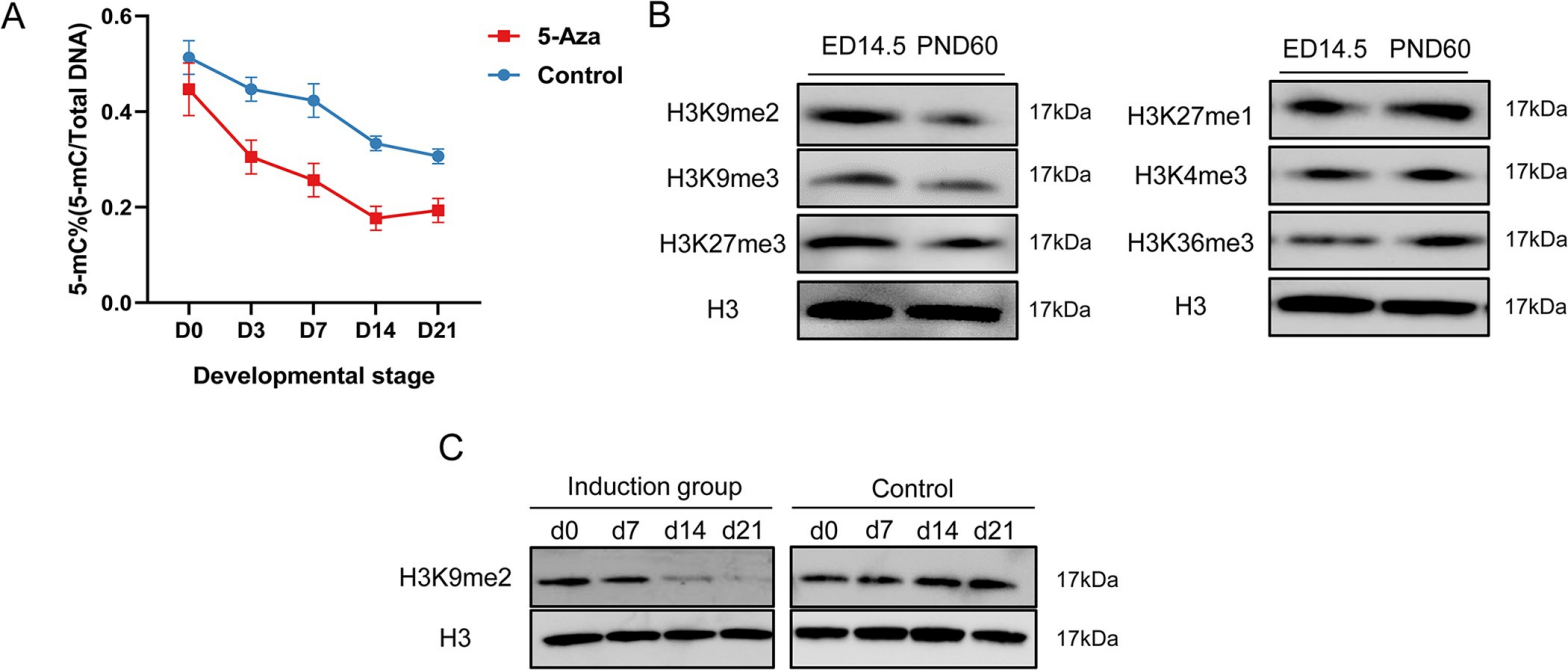
Identification of epigenetic influences on the differentiation process of BMSCs. (A) Changes in the content of 5mC in genomic DNA during the induction of BMSCs were detected by ELISA; the control group was the self-differentiated group, ** *p* <0.01 (0–21 days). (B) Detection of changes in methylation of key histones in cardiomyocytes in the hearts of rats at embryonic day 14.5 and 60 days after birth using Western blot analysis. (C) the change trend of H3K9me2 during induction differentiation, with H3 as internal control.

To examine changes in the global DNA methylation status of the cellular genome during BMSC differentiation, we evaluated changes in 5mC content by ELISA, measuring the levels during the course of induction for up to 21 days. The results showed that the naturally differentiated group experienced a gradual decline in 5mC content during the induction process(*p* <0.01) (Fig 1A), indicating that the progression towards cardiomyocyte-like cell formation was accompanied by a slow decrease in DNA methylation. This decline was significantly accelerated using DNA methylation inhibitors, which further effectively reduced global genomic 5mC content and total cellular DNA methylation levels.

To investigate the potential significance of histone methylation in cardiomyocyte differentiation, we evaluated changes in the levels of key related histone methylation marks present in cardiomyocytes derived from rat hearts at 14.5 days gestational age and 60 days postpartum. The levels of H3K27me3, H3K9me3/me2, H3K27me1, H3K4me3 and H3K36me3 in myocardium at different ages were detected by Western blotting. Our findings indicated that H3K27me3 and H3K9me3/me2 significantly decreased, whereas H3K27me1, H3K4me3 and H3K36me3 showed slight increases at PND60. Of all the histone modifications examined, the most significant change was observed in H3K9me2 (Fig 1B). Similarly, when BMSCs were induced to differentiate using TBX18 in vitro, the overall level of H3K9me2 was found to gradually decrease during the induction process from 0–21 days (Fig 1C). The above results indicate that H3K9me2 is a critical histone methylation involved in the differentiation process of BMSCs.

### Differential enrichment of DNA methylation and H3K9me2 in the promoter region of the key gene HCN4 in pacemaker cells

**Fig 2:**
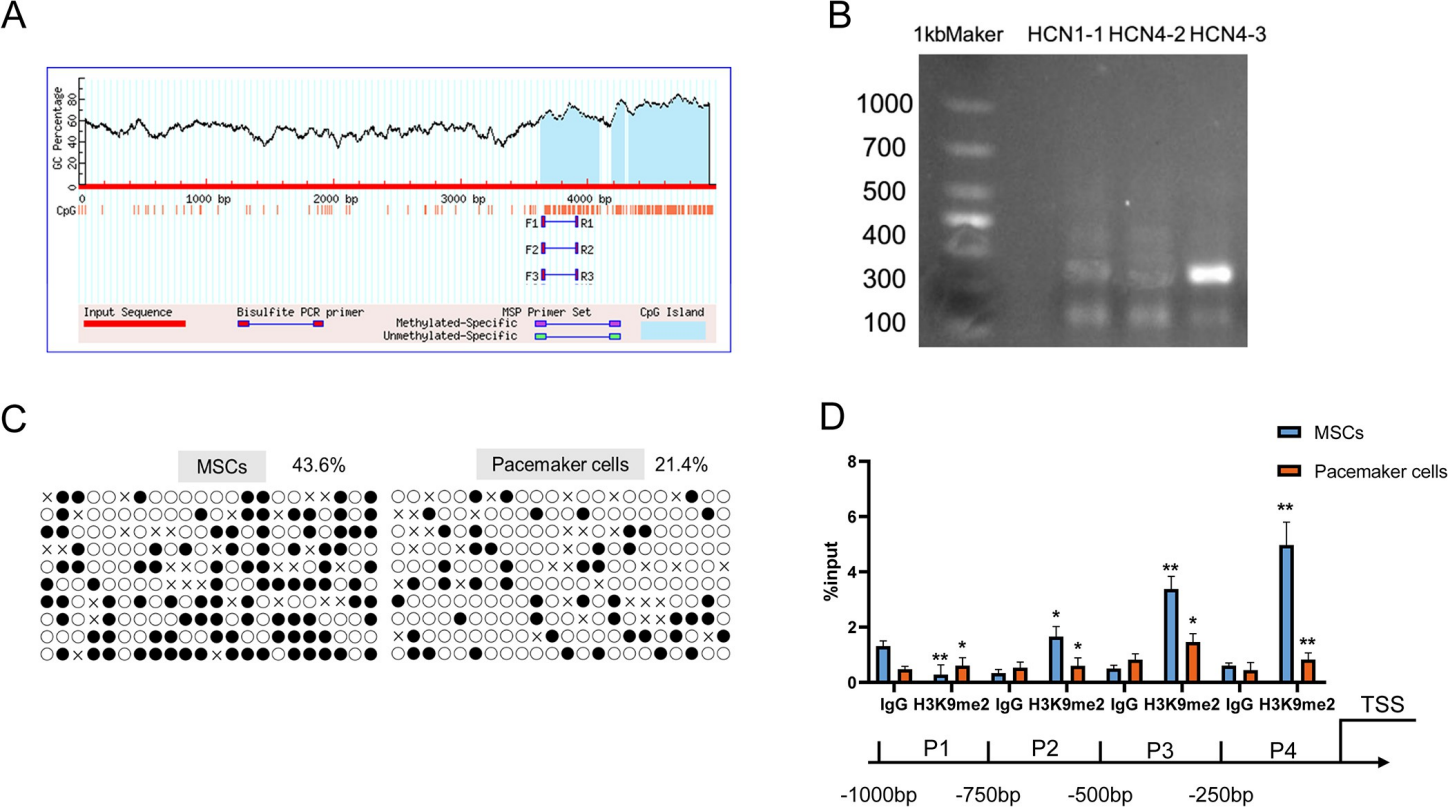
DNA methylation and H3K9me2 analysis of HCN4 promoter region. (A) HCN4 promoter region CpG island prediction and primer design. (B) CpG islands in the HCN4 promoter region were specifically amplified by three different primers after bisulfite transformation. (C) Detection of DNA methylation rate in the promoter region of the HCN4 gene in BMSCs and Pacemaker cells. Note: Each circle represents a CG site, black represents methylation occurred, white represents unmethylation, each group has 10 rows respectively, representing 10 replicates. (D) ChIP-qPCR to detect enrichment of H3K9me2 near the core promoter region of HCN4. Compared with total protein pulled by IgG antibody, * *p* < 0.05 and ** *p* < 0.01.

Furthermore, we analysed and compared the changes in DNA methylation levels of the key HCN4 promoter between pacemaker cells and BMSCs, and three CpG islands were successfully predicted by Methprimer in the HCN4 promoter region (5 kb upstream of the transcription start site, Fig 2A). Using the designed primers, we amplified the transformed DNA and found that the HCN4-3 primer could amplify clear bands, which was then selected as the amplification primer for subsequent experiments (Fig 2B). After comparison with the original sequence, we discovered that the HCN4 promoter region in pacemaker cells showed significantly lower levels of DNA methylation than that in BMSCs (Fig 2C, *p* < 0.05). Similarly, to verify the changes in H3K9me2 levels at the HCN4 gene promoter, we divided the promoter region upstream of the TSS into four equal regions, based on the core HCN4 promoter region (267bp upstream of TSS) identified by previous authors [25]. Using ChIP-qPCR, we found that H3K9me2 was virtually enriched in regions P2-P4 (0-750bp upstream of TSS), with particularly significant enrichment in region P4 (0-250bp upstream of TSS) (Fig 2(D), *p* < 0.01).

### Inhibition of DNA and H3K9 methylation increased the efficiency of transduction of TBX18 to induce the formation of pacemaker cells

**Fig 3:**
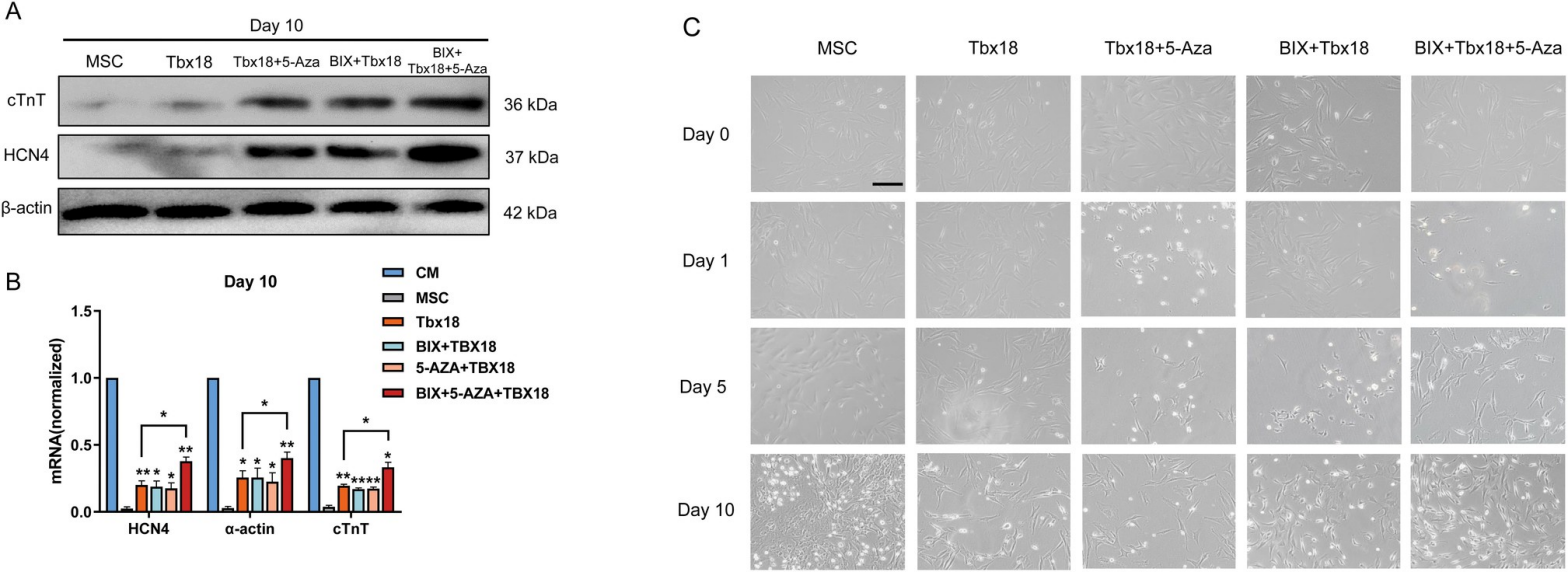
Combination of DNA and histone methylation inhibitors to optimize TBX18 induction system. (A) Western blot was performed to detect HCN4 and cTnT protein expression. (B) qRT-PCR analysis of HCN4, α-actin and cTnT mRNA expression levels. (C) Morphological observation of the cellular state of BMSCs after days 0, 1, 5 and 10 of different treatments. Scale bar = 0 μm.

To verify the effects of DNA methylation and histone methylation on differentiation induced by Tbx18 transduction in BMSCs, BIX01294 and 5-Aza were used in combination in the Tbx18-BMSCs system. qRT-PCR and Western blotting results showed that after 10 days of single treatment with 5-Aza and BIX01294, the gene expression of α-actin, cTnT, and HCN4 in BMSCs was significantly higher than that of the untreated group (Fig 3A and 3B). However, both mRNA and protein expression reached the highest level after the combined addition of both, and were significantly higher than in the transduced Tbx18 group without additives (Fig 3A and 3B). Morphological observations also showed that the number of sinoatrial node cells proliferated the most at day 10 (Fig 3C), and the number of beating cells reached 35%. These results demonstrated that DNA methylation and BIX01294 could regulate the formation of pacemaker cells, and the combination of 5-Aza and BIX01294 can further optimise the system of transduced Tbx18 to induce the differentiation of BMSCs into pacemaker-like cells.

### DNA methylation and H3K9me2 mediated transcription factor regulation of the initiation activity of the key gene HCN4

**Fig 4:**
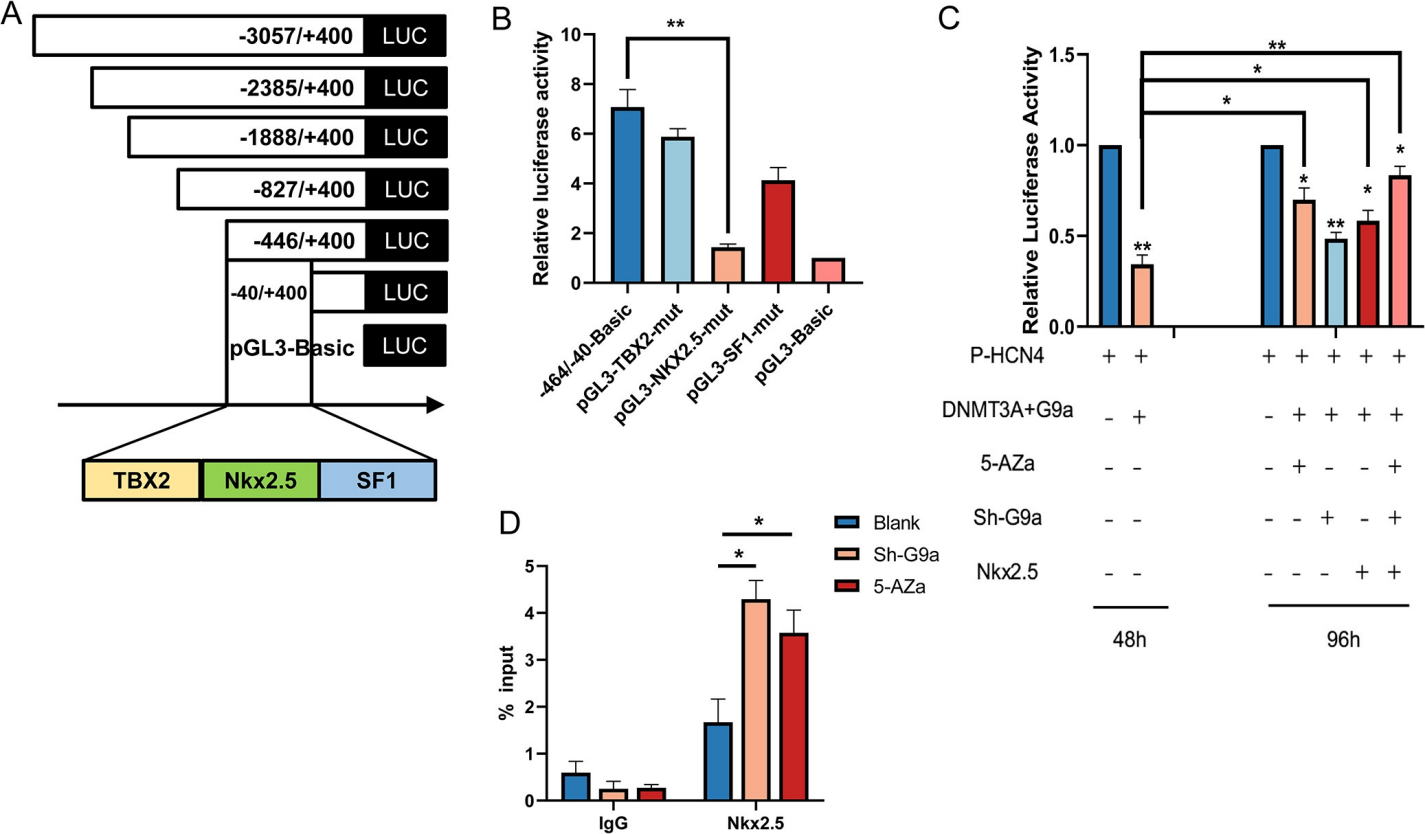
Effect of epigenetic factors on the transcriptional activity of the HCN4 promoter region. (A) Prediction of transcription factors in the core promoter region of HCN4. (B) Dual luciferase reporter system detects promoter fragment activity after deletion of the corresponding binding site. (C) Detection of DNA methylation and the effect of H3K9me2 and transcription factor NKx2.5 on HCN4 initiation activity by a dual luciferase reporter system. (D) ChIP-qPCR detects NKx2.5 binding changes in the HCN4 promoter region. * *p* < 0.05 and ** *p* < 0.01.

Since the HCN4 gene promoter was observed to be co-regulated by DNA methylation and histone methylation during the formation of pacemaker cells, to further analyse the transcriptional regulatory mechanism of HCN4, we predicted the potential transcription factors NKx2.5, Tbx2, and SF1 in the core promoter region of HCN4 using JASPAR (Fig 4A). After deletion of the corresponding binding sites of the three factors, the dual luciferase reporter system showed that the promoter activity decreased, but the most significant decrease in initiation activity was observed after deletion of NKx2.5 (Fig 4B, *p*<0.01), indicating that it belongs to the key fragment of the promoter region. Thus, we confirmed that NKx2.5 is the key transcription factor of HCN4.

Based on the above results, to further verify how DNA methylation, H3K9me2, and transcription factors mutually regulate the initiation transcription of HCN4, we designed transfection experiments with different combinations. First, we cotransfected BMSCs with DNMT3A and G9a overexpression vectors and p-HCN4base vector, and the results showed that the initiation activity of HCN4 was significantly reduced (Fig 4C, *p*<0.05). Then, after 48 h, 5-Aza and shG9a or OE-NKx2.5 vectors were added separately, and after 96 h, we detected a significant rescue of the promoter activity compared to before. When 5-Aza was added and shG9a and OE-NKx2.5 were co-transfected, the promoter activity increased significantly and reached its highest level (Fig 4C, *p*<0.01). These results suggest that the HCN4 promoter activity is dependent on the co-regulation of DNA and histone methylation and transcription factors, where DNA and H3K9me2 methylation have a negative regulatory effect and the NKx2.5 transcription factor has a positive activating effect.

To date, we have speculated whether these two methylation patterns affect the binding of the transcription factor NKx2.5 in the HCN4 promoter region, thereby indirectly regulating the transcription of HCN4 expression. Therefore, we analysed the binding of NKx2.5 to the HCN4 promoter region in BMSCs under different treatments using ChIP-qPCR. We found that the binding of NKx2.5 on HCN4 significantly increased after 5-Aza treatment (Fig 4D, *p*<0.05), and the binding of NKx2.5 also significantly increased after G9a interference (Fig 4D, *p*<0.05). These results indicate that the binding of NKx2.5 in the HCN4 promoter region is indeed inhibited by DNA methylation and H3K9me2.

### Inter-coupling of DNA methylation and H3K9me2 in the HCN4 promoter region

**Fig 5:**
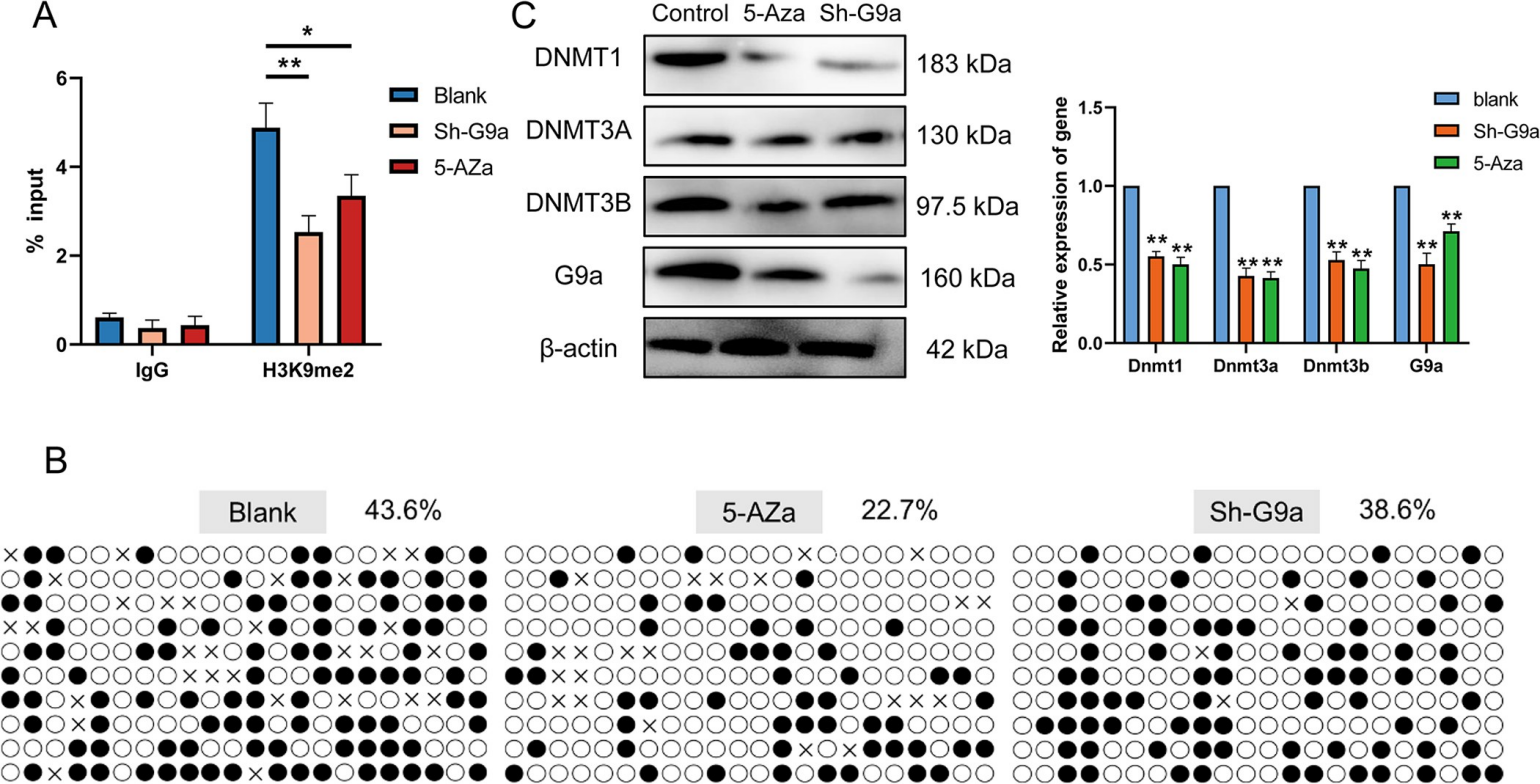
Validation of DNA methylation and H3K9me2 HCN4 in the promoter region interaction relations. (A) ChIP-qPCR detects the enrichment levels of H3K9me2 in the vicinity of the HCN4 promoter region. (B) BSP detects DNA methylation status of CG sites in HCN4 promoter region after different treatments. (C) Detection of DNA and histone methylation enzyme expression changes by qRT-PCR and Western-blot. * *p* < 0.05 and **p < 0.01.

It remains unclear how DNA methylation and H3K9me2 specifically regulate the transcriptional activation of HCN4 and whether there is any correlation or influence between the two epigenetic modifications. Therefore, we first validated the interaction mechanism by inhibiting one methylation pattern and analysing changes in the other methylation pattern and methyltransferase. After transfecting BMSCs with shG9a or treating them with 5-Aza, we detected a significant decrease in the enrichment level of H3K9me2 near the core promoter region of HCN4 in G9a-interfered BMSCs by ChIP-qPCR (Fig 5A), while a significant decrease was also observed in the 5-Aza-treated group compared to the control group (Fig 5A). This indicates that both histone methyltransferase interference and the addition of DNA methylation inhibitors can down-regulate the binding level of H3K9me2 at the HCN4 promoter region.

Next, we detected changes in DNA methylation levels in the HCN4 promoter region in the BMSCs group after different treatments by bisulfite sequencing PCR (BSP). After amplification and alignment to the original sequence after bisulfite treatment, we found that the DNA methylation level at the promoter region of the HCN4 gene showed a statistically significant decrease in cells treated with 5-Aza (Fig 5B, *p*<0.05), while there was no significant change in the G9a interference group. These results suggest that DNA methylation inhibitors can reduce the level of DNA methylation in the promoter region of the HCN4 gene to some extent, whereas interference with G9a methyltransferase has no effect.

Further, we observed that the mRNA and protein levels of DNA methyltransferases (DNMT1, DNMT3A, and DNMT3B) and histone methyltransferase G9a decreased after the addition of 5-Aza. Similarly, interference with G9a also led to a decrease in both DNA methyltransferases and G9a, with DNMT1 showing the most significant reduction (Fig 5C, *p*<0.01), while the expression changes of DNMT3A and DNMT3B were slightly less. The above results indicate that DNA methylation and H3K9me2 are in a coupled relationship and the two methylesterases can influence each other.

### G9a and DNMT1 form a complex that modifies the epigenetic HCN4 promoter region

**Fig 6:**
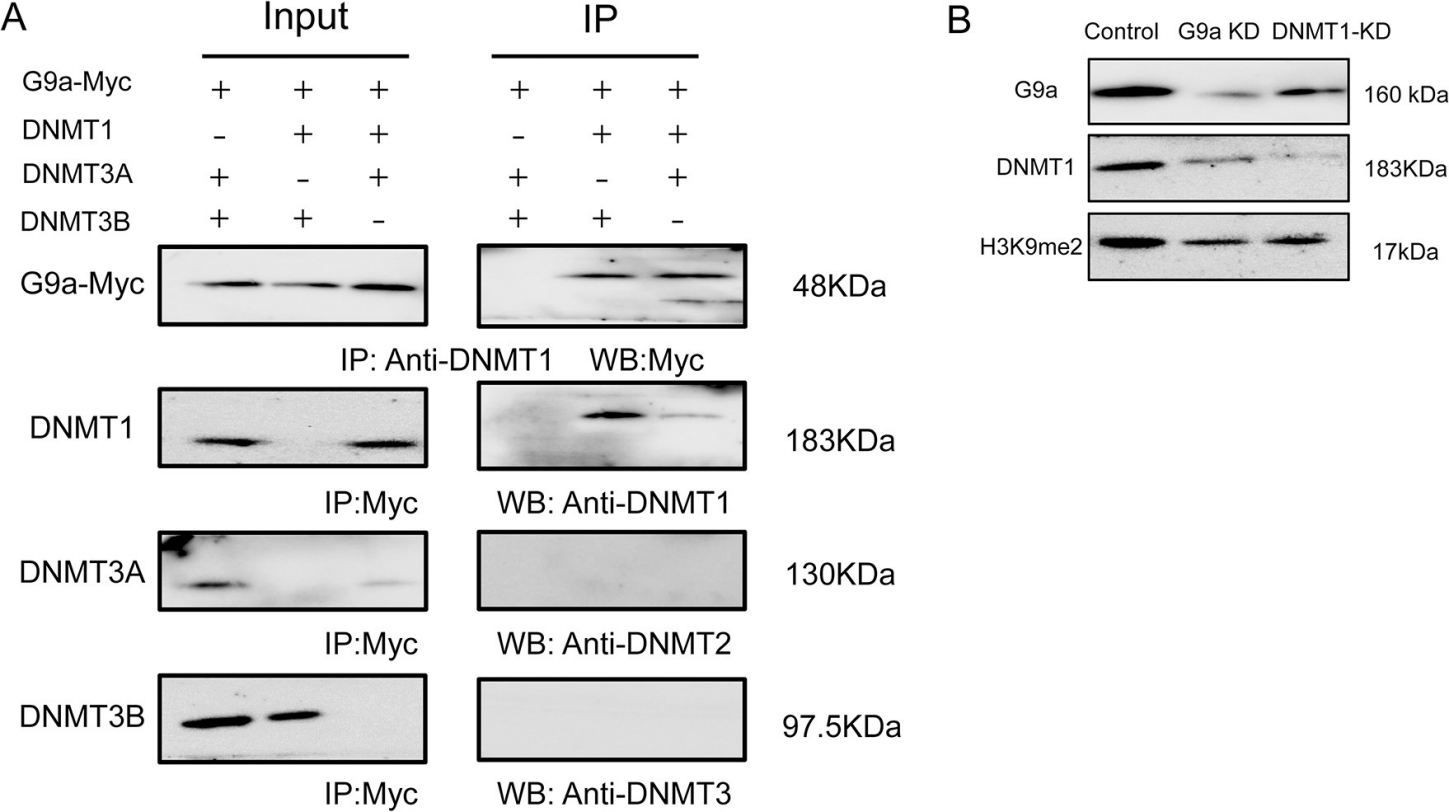
Interaction between G9a and DNMT1. (A) Co-immunoprecipitation of G9a and DNMT1/3A/3B in cell extracts from BMSCs cells transfected with G9a-Myc and DNMT1/3A/3B constructs. (B) Western blot analysis of G9a, DNMT1 and H3K9me2 levels after knockdown of G9a and DNMT1, respectively.

To clarify the specific binding relationship between DNA methylesterase and G9a, we co-transfected the G9a-Myc fusion expression vector with DNMT1, DNMT3A and DNMT3B overexpression vectors into BMSCs. After 48 hours, we immunoprecipitated protein complexes from cell extracts using Myc and DNMT enzyme antibodies, and found that both G9a-Myc and DNMT1 were pulled down by Myc and DNMT1 antibodies in the co-transfection group of DNMT1 and G9a fusion expression vectors by Western blotting. However, in the co-transfection group of DNMT3A and DNMT3B with G9a-Myc, Myc antibodies were unable to pull down DNMT3A/B proteins, and DNMT3A/B antibodies also failed to pull down G9a-Myc (Fig 6A). This suggests that there is a direct interaction between DNMT1 and G9a in BMSCs cells.

There is a certain synergistic effect between DNA methylation and H3K9 methylation in the silencing of gene expression. To verify the mutual influence between DNMT1 and G9a in mediating DNA methylation and H3K9 methylation, we performed shRNA-mediated knockdown expression of G9a and DNMT1 in BMSCs and found that knockdown of G9a resulted in a significant decrease in H3K9me2 and DNMT1 in cell extracts and chromatin in the HCN4 promoter region, while knockdown of DNMT1 also resulted in a decrease in G9a accompanied by a reduction in H3K9me2 levels (Fig 6B). These results suggest that G9a and DNMT1 may recruit each other to form a complex in the HCN4 promoter region, thereby regulating the methylation status of chromatin.

## Discussion

Our research revealed the critical role of the direct interaction between H3K9 and DNA methylation in the formation of pacemaker-like cells in rats through the regulation of HCN4. We found significant differences in DNA methylation and H3K9me2 levels on the HCN4 promoter region in MSCs and pacemaker cells, and successfully screened to identify the key transcription factor NKx2.5 in the promoter region. In vitro experiments showed that DNA methylation and H3K9me2 as well as the transcription factor NKx2.5 could affect the initiation activity of HCN4 to some extent. Importantly, DNA methylation and H3K9me2 are coupled under the regulation of methylation enzymes, DNMT1 and G9a bind in a complex to regulate both H3K9 and DNA methylation in the promoter region of HCN4, and the two epigenetic marks cooperate to silence transcription.

DNA methylation is a key regulator of the specific differentiation of MSCs into functional cells. DNA methylation during the differentiation of porcine MSCs into adipocytes shows dynamic changes accompanied by changes in the expression pattern of DNA methyltransferase enzymes [26]. We have also observed that the self-differentiation process of BMSCs is accompanied by a slow decrease in DNA methylation levels, and that 5-Aza can replace cytosine and covalently bind to DNMT, thus blocking DNA methylation replication [27, 28]. Therefore, we observed a sustained decrease in genomic DNA methylation levels during 5-aza-induced differentiation of BMSCs into cardiomyocytes. In addition to DNA methylation, histone methylation is also an important epigenetic mark, especially the methylation of H3K9 and H3K27 catalysed by G9a and EZH2 respectively are repressive markers [29]. Our results show significant differences in H3K9 methylation in rat cardiomyocytes and BMSCs, but not in H3K27 methylation, which may be related to the different cellular models. In recent years, some investigators have demonstrated that H3K9 methylation plays an important role in cardiac remodelling [30] and that by regulating H3K9me2 can affect, it the expression of genes related to cardiac development [31].

Compared to gene regulatory elements, epigenetic modifications can respond dynamically to external environmental signals and regulate gene expression by interacting with transcription factors. For example, DNA methylation on cytosines within CpG dinucleotides can silence gene expression by altering chromatin structure and preventing transcription factors from binding to it [32–34]. Histone modifications also affect chromatin structure, which is crucial for the interaction between transcription factors and promoter elements, such as acetylation of histones H3 and H4 or trimethylation on lysine 4 (H3K4me3), which activates transcription by loosening chromatin structure and allowing recruitment of transcription factors [35, 36]. In this study, we also found that the transcription factor NKx2.5 was inhibited by DNA methylation and H3K9me2 binding in the HCN4 promoter region, and that release of this inhibition activated HCN4 expression.

As the study of epigenetic mechanisms progresses, researchers are increasingly interested in the correlation and mechanisms of interaction between different methylation modification patterns in the epigenetic field. In vivo studies have confirmed certain molecular links between the mechanisms that maintain DNA methylation and H3K9 methylation, for example, research in Arabidopsis thaliana suggests that DNA methylation is strictly dependent on the H3K9 methyltransferase [37]. However, the lack of H3K9 methylating enzymes in mammals has a more complex effect on DNA methylation at endogenous reverse transcription sites [38]. Previous studies have shown that treatment with 5-Aza leads to a decrease in DNA methylation levels, loss of H3K9 methylation, and an increase in H3K4 methylation, which is similar to the results observed in our study [39]. DNMT1 has been shown to bind directly to G9a both in vitro and in vivo and to colocalise in the nucleus during DNA replication, thereby co-ordinating DNA and H3K9 methylation during cell division [40]. The heteromeric G9a/GLP complex has been reported to regulate both H3K9 and DNA methylation, contributing to the cooperative silencing of transcription by both epigenetic marks [41]. This also explains why we observed that knockdown of G9a led to a reduction in DNA methyltransferase activity, in particular a sharp decrease in DNMT1 expression levels, which are responsible for maintaining DNA methylation.

Importantly, in vitro experiments showed that inhibition of DNA and H3K9 methylation can significantly improve the efficiency of inducing pacemaker-like cells by transducing TBX18, which holds promise for optimising in vitro pacemaker cell induction protocols.

## Conclusion

In conclusion, these results provide the first evidence that DNMT1 and G9a complexes mediate the mutual coupling of DNA and H3K9 methylation, which plays a critical role in regulating the differentiation of MSCs into pacemaker-like cells by inhibiting the transcription factor NKx2.5 to regulate HCN4 transcription.

## Supporting information

S1 Data (XLSX)

S1 Raw images (PDF)
