# Supplementary material for: DNA methylation cooperates with H3K9me2 at HCN4 promoter to regulate the differentiation of bone marrow mesenchymal stem cells into pacemaker-like cells
Source: PLoS One. 2023 Aug 29;18(8):e0289510. doi: 10.1371/journal.pone.0289510 (PMC10464974; doi:10.1371/journal.pone.0289510)
Supplement: S1 Raw images — (PDF) [file pone.0289510.s002.pdf]

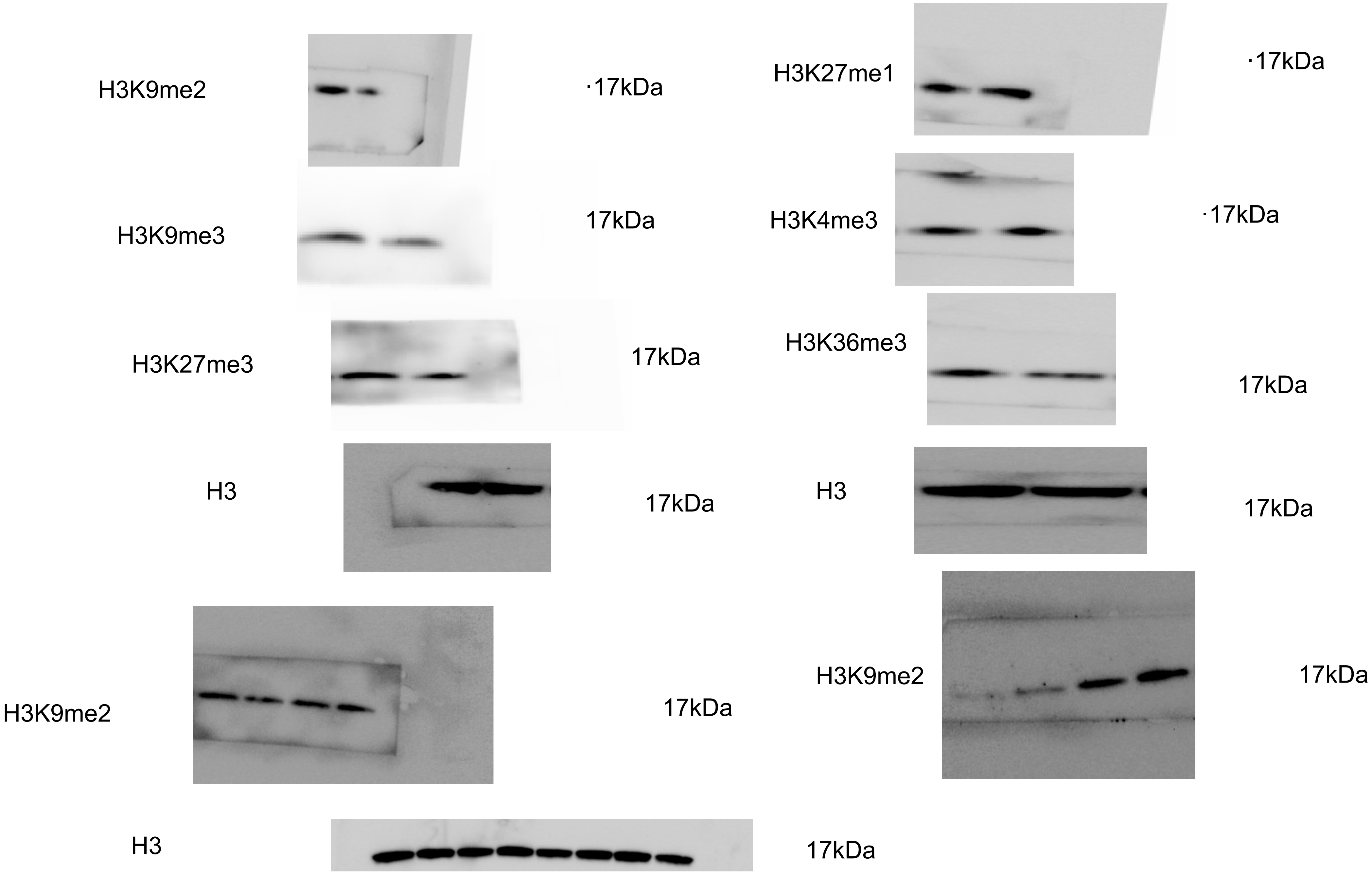
H3K9me2
·17kDa
H3K9me3
17kDa
H3K27me3
17kDa
H3
17kDa
H3K9me2
17kDa
H3
17kDa
H3K27me1
·17kDa
H3K4me3
·17kDa
H3K36me3
17kDa
H3
17kDa
H3K9me2
17kDa

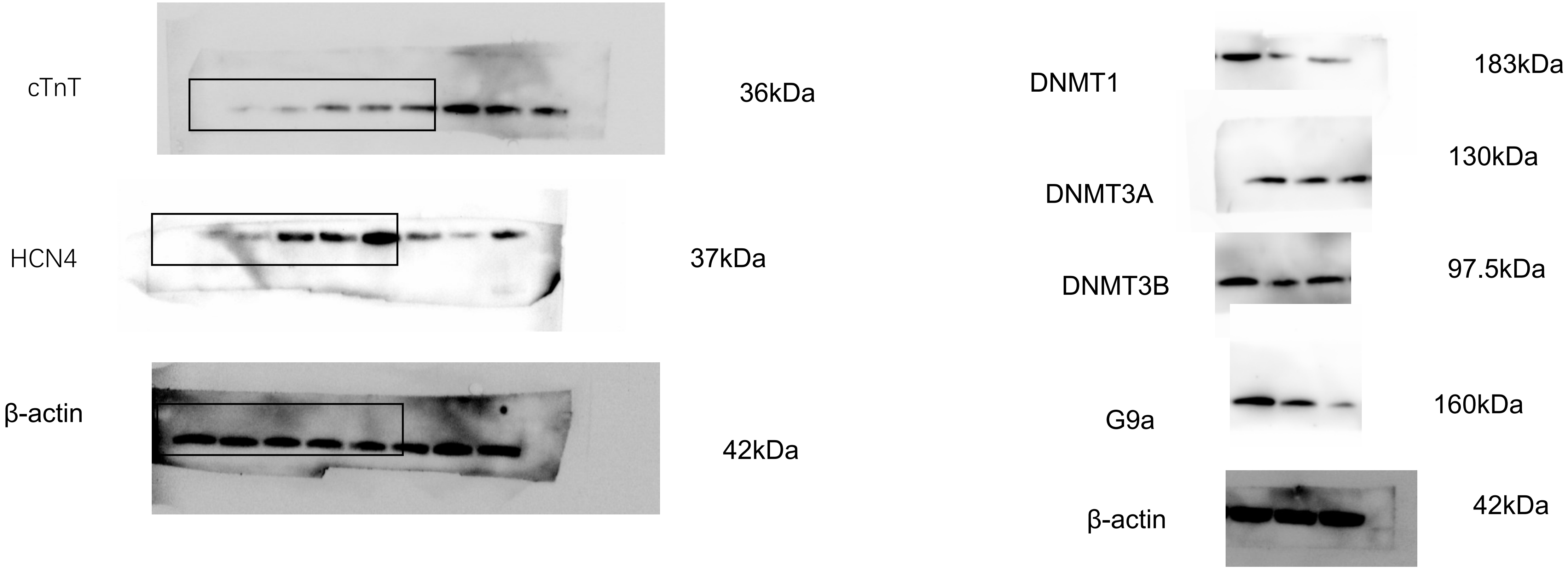
cTnT
36kDa
HCN4
37kDa
β-actin
42kDa
DNMT1
183kDa
DNMT3A
130kDa
DNMT3B
97.5kDa
G9a
160kDa
β-actin
42kDa

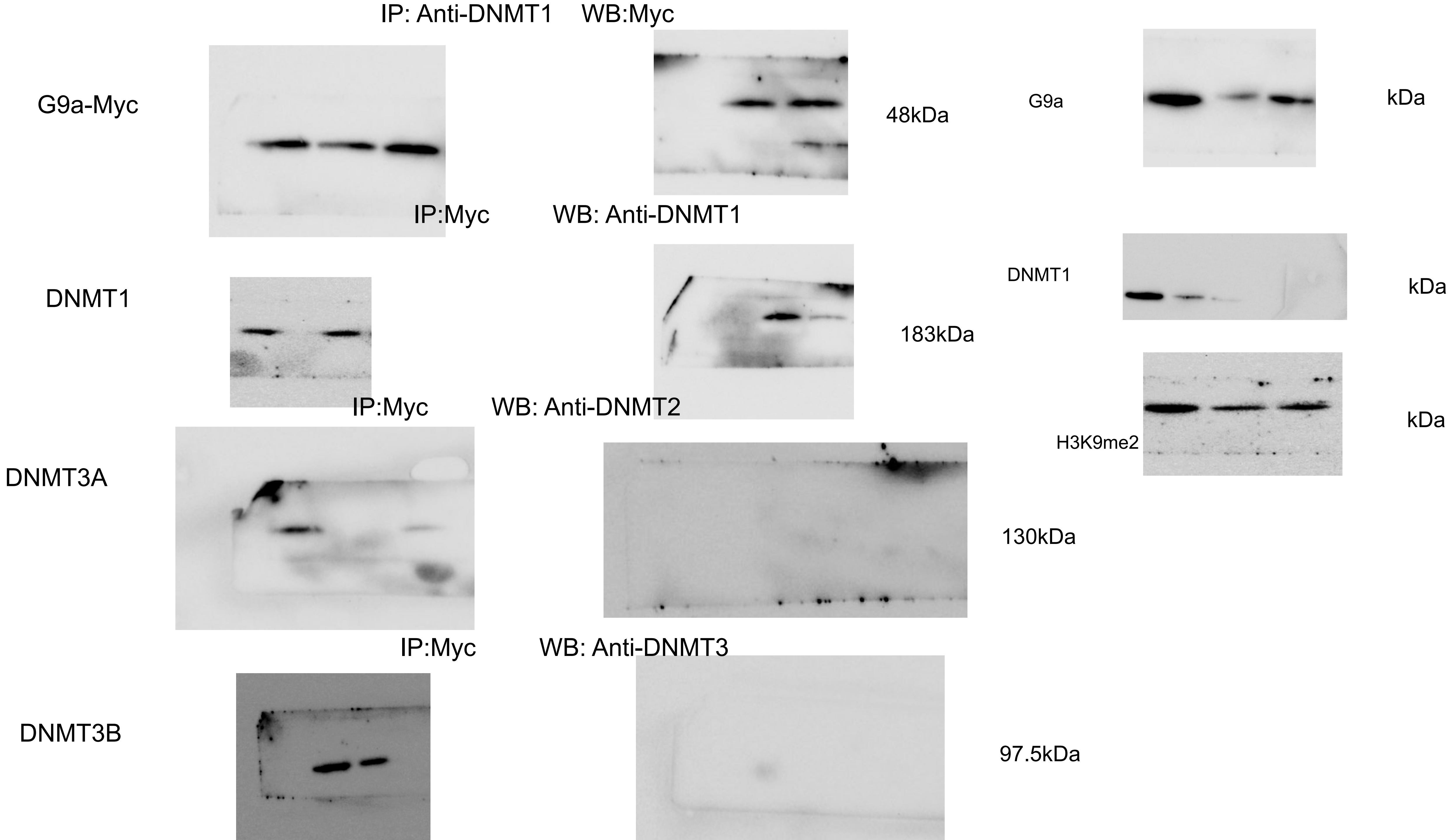
IP: Anti-DNMT1 WB:Myc
G9a-Myc
48kDa
G9a
kDa
IP:Myc WB: Anti-DNMT1
DNMT1
183kDa
DNMT1
kDa
H3K9me2
kDa
IP:Myc WB: Anti-DNMT2
DNMT3A
130kDa
IP:Myc WB: Anti-DNMT3
DNMT3B
97.5kDa
